# An Improved Manufacturing Approach for Discrete Silicon Microneedle Arrays with Tunable Height-Pitch Ratio

**DOI:** 10.3390/s16101628

**Published:** 2016-10-09

**Authors:** Renxin Wang, Wei Wang, Zhihong Li

**Affiliations:** 1 Science and Technology on Electronic Test & Measurement Laboratory, North University of China, Taiyuan 030051, China; 2 National Key Laboratory of Science and Technology on Micro/Nano Fabrication, Institute of Microelectronics, Peking University, Beijing 100871, China; wei_w77@163.com; 3 Key Laboratory of Instrumentation Science & Dynamic Measurement, Ministry of Education, North University of China, Taiyuan 030051, China

**Keywords:** discrete, microneedle, KOH etching, octagonal pyramid

## Abstract

Silicon microneedle arrays (MNAs) have been widely studied due to their potential in various transdermal applications. However, discrete MNAs, as a preferred choice to fabricate flexible penetrating devices that could adapt curved and elastic tissue, are rarely reported. Furthermore, the reported discrete MNAs have disadvantages lying in uniformity and height-pitch ratio. Therefore, an improved technique is developed to manufacture discrete MNA with tunable height-pitch ratio, which involves KOH-dicing-KOH process. The detailed process is sketched and simulated to illustrate the formation of microneedles. Furthermore, the undercutting of convex mask in two KOH etching steps are mathematically analyzed, in order to reveal the relationship between etching depth and mask dimension. Subsequently, fabrication results demonstrate KOH-dicing-KOH process. {321} facet is figured out as the surface of octagonal pyramid microneedle. MNAs with diverse height and pitch are also presented to identify the versatility of this approach. At last, the metallization is realized via successive electroplating.

## 1. Introduction

Recently, microneedle arrays (MNAs) draw more and more attention due to their great prospective in various applications such as transdermal electroporation [1], transdermal drug delivery [2], dry biopotential electrodes [3] and penetrating neural electrodes [4]. Flexible penetrating electrodes are preferred to adapt to curved and elastic tissue [4]. The contradiction is that penetration requires rigid microneedles whereas flexible substrates are also needed. Hence, discrete MNAs, which means the microneedles are different from the substrate in terms of their materials, are needed to fabricate this kind of flexible penetrating electrode via a transferring method. Diverse MNAs utilizing versatile processes, materials and shapes have been proposed, as shown in Table 1. Biodegradable materials such as polylactic acid (PLA) [5], poly-lactic-co-glycolic acid (PLGA) [6], polycaprolactone (PCL) [7], interferon α and polyvinyl alcohol (PVA) [8], sugar glass [9] and non-biodegradable polymer SU-8 [10] have been applied in molding-based fabrication of MNAs. The substrate and microneedles are usually made of the same material used in the molding process, otherwise the adherence between substrate and microneedle is too weak to maintain the MNA. Ren developed PLGA-based and Ti/Au-coated hill-like MNAs fabricated by thermal drawing for bio-signal monitoring [3]. 

The backside inadequate exposure technique was introduced to form SU-8 MNAs [11]. The electron discharge machining (EDM) method is presented to manufacture stainless steel MNAs [12]. These approaches could be considered as reductive manufacturing of the substrate. Hence, these MNAs have poor/fair discreteness, meaning that the microneedles could not be separated with the substrate in terms of material. Electrochemical etching titanium and microneedle insertion are performed to assemble titanium MNAs [13], which possess discrete microneedles but offer poor mass production possibilities. 

On the other hand, silicon is widely used in fabricating MNAs due to its versatile tailoring process and high tensile strength, compared with other microneedle materials [14]. HNA (a mixture of HNO_3_, HF and HOAc) etching could be applied on silicon pillars via deep reactive ion etching (DRIE) or dicing in order to sharpen pillars until microneedles are formed [2,15,16]. However, HNA solution is so corrosive that heterogeneous substrates would be dramatically etched. Consequently, homogeneous silicon substrates are commonly adopted. Self-stabilized diamond-shaped microneedle formation via 2 stage etching was demonstrated [17]. Its base is very narrow and might be not strong enough to stand alone on a non-rigid heterogeneous substrate. A kind of discrete nipple-shaped microneedle prepared via dry etching was proposed, yet its uniformity is fair [4]. An octagonal pyramid silicon MNA was obtained via one stage KOH etching, which provided discrete and uniform microneedles. However, the ratio of height to pitch is very low (only 0.25), which is equivalent to density as the height is certain. As a matter of fact, it would reach a ceiling limit of about 0.46 according to the analysis of Section 3 (Equation (5)). Therefore, an alternate method is proposed in order to fabricate discrete MNA with tunable height-pitch ratios, which involves a KOH-dicing-KOH process.

## 2. Fabrication Process

N(100) silicon wafer with resistivity of 2~4 Ω·cm is adopted as target object and a KOH etching process is kept in the condition of 30% concentration and 80°C temperature when mechanical agitation is applied. The fabrication process mainly consists of three steps: first KOH etching, dicing and second KOH etching. Specifically, the process is started from bonding a silicon wafer to glass, with patterned Si_3_N_4_ as KOH mask composing of square and strip. Then, first KOH etching is performed to thin down the bulk silicon. The wafer is diced along the middle line of two adjacent square masks.

Dicing depth is well designed to make sure silicon with a certain thickness is retained. Silicon pillars with cross-like mask are formed. Eventually, second KOH etching is brought about. Owing to KOH undercutting of the convex mask corner, the silicon pillar would be etched along the facet with fast etching rate and finally silicon needles come into being after the cross-like mask is thoroughly undercut. It should be pointed out that the retained silicon during the dicing process is also etched just right, to obtain discrete silicon MNAs.

**Figure 1:**
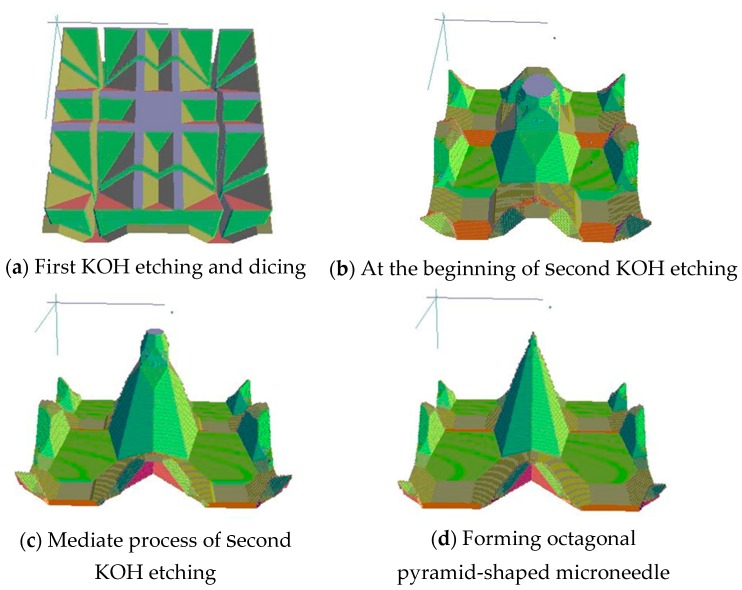
Simulation of second KOH etching.

Here, KOH-dicing-KOH process is simulated via Anisotropic Crystalline Etching Software (ACES). It should be noted that there is no model for the dicing process in ACES. The simulation of dicing is replaced with that of deep reactive ion etching (DRIE). The simulation results are illustrated in Figure 1: (a) silicon pillars are formed after first KOH etching and dicing; (b) Second KOH etching is started and the strips disappeared; (c) as the etching process goes on, the square mask begins to be undercut; (d) finally, the mask is thoroughly undercut and the octagonal pyramid-shaped microneedle comes out.

## 3. Mathematical Model

Two KOH etching processes are mathematically concerned. Generally, undercutting happens beneath convex corner mask and along the inclining facet with fast etching rate. For KOH etching with 30% concentration and 80 °C temperature, the inclining facets are the cluster of {411} facets. As the mask undercutting is prominently concerned, the evolution of <410> crystal orientation is illustrated, which is the intersection line of {411} facet and {100} facet. The analyses could be divided into two parts.

### 3.1. First KOH Etching

**Figure 2:**
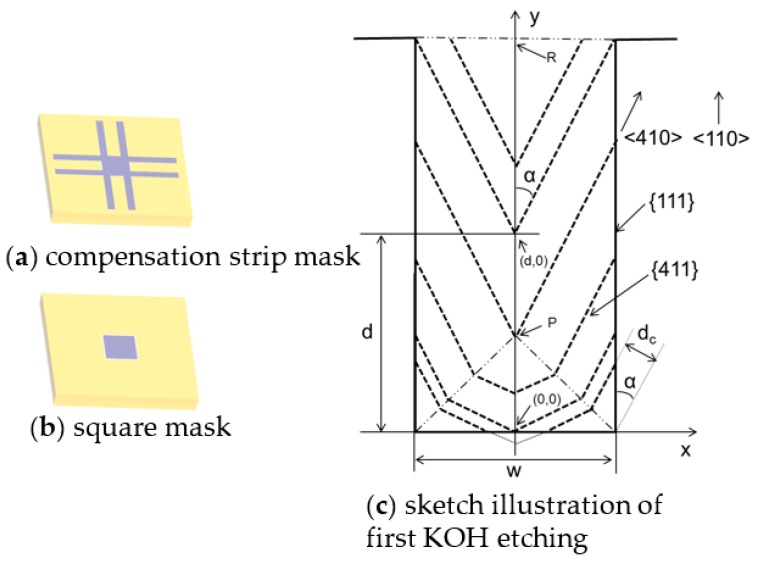
Sketch illustration of successive lateral etching beneath compensation strip. (First KOH etching).

First KOH etching starting from concentration strip mask is analyzed, as in Figure 2. Width of strip is *w*, angle of <410> and <110> is *α* = 31.0°, the distance from starting point to <410> is *d_C_*, the interception of inclining facet is *d*. It could be deduced that:

(1) $$d_{c}=0.5w\sin\alpha +d\cos\alpha -0.5w\tan\alpha \leq d\leq 0.5w$$

(2) $$d_{c}=0.5w\cos\alpha +d\sin\alpha \;d\geq 0.5w$$

The angle of {411} and {100} is *β* = 76.4°. $V_{\text{\{}411\text{\}}}/V_{\text{\{}100\text{\}}}=1.46$, where *V*_{100}_ and *V*_{411}_ are etching rates of {100} and {411} respectively. The evolution rate of *d_C_* is $V_{d_{c}}=V_{\text{\{}411\text{\}}}/\sin\beta$. Hence, the etching depth of *H* could be figured out:

(3) $$H=\frac{d_{C}}{V_{d_{c}}}V_{\text{\{}100\text{\}}}=d_{C}\sin\beta \frac{V_{\text{\{}100\text{\}}}}{V_{\text{\{}411\text{\}}}}$$

For concentration strip mask with width of *W_c_* and length of *L* ($L\geq 0.5W_{C}$), its maximum etching depth *H_cmax_* could be derived from Equations (2) and (3):

(4) $$H_{C\max}=(0.5W_{C}\cos\alpha +L\sin\alpha )\sin\beta \frac{V_{\text{\{}100\text{\}}}}{V_{\text{\{}411\text{\}}}}=0.3W_{C}+0.36L$$

Similarly, square mask could be considered as two concentration strip mask, where etching starts from both side. For square mask with width of *W_S_*, it would disappear when *d_max_ = 0.5W_S_*. Maximum etching depth *H_Smax_* could be derived from Equations (1) and (3):

(5) $$H_{S\max}=(0.5W_{S}\sin\alpha +d_{\max}\cos\alpha )\sin\beta \frac{V_{\left\{100\right\}}}{V_{\left\{411\right\}}}=0.46W_{S}$$

### 3.2. Second KOH Etching

**Figure 3:**
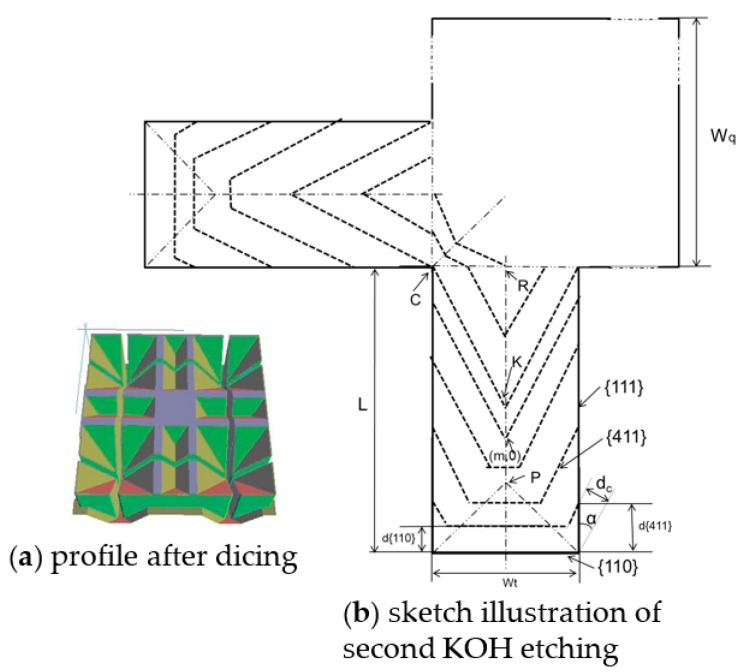
Sketch illustration of successive lateral etching beneath compensation strip. (After dicing, second KOH etching).

As for second KOH etching, the situation is different, as shown in Figure 3. The mask consists of square mask with width of *W_q_* and concentration strip with *L* length and *W_q_* width. The beginning facet for strip undercutting is {110} created by dicing rather than {100}. 

Considering *V*_{110}_:*V*_{411}_ *= 1*, the etchings of {110} and {411} simultaneously proceed. When {411} facet arrives point C, square mask starts to be undercut. The undercutting of strip goes on until {411} facet arrives point R. The overall etching depth along {100} is *H_j_*. *H_ct_* is the etching depth during the period when strip is undercut, *H_sq_* is the depth related to square mask, *H_d_* is the depth during the time from point C to R:

(6) $$H_{j}=H_{\mathrm{ct}}+H_{sq}-H_{d}=[0.5W_{t}\cos\alpha +L\sin\alpha +0.5W_{q}(\sin\alpha +\cos\alpha )-0.5W_{t}\sin\alpha ]\sin\beta \frac{V_{\left\{100\right\}}}{V_{\left\{411\right\}}}=0.13W_{t}+0.36L+0.46W_{q}$$

Considering *V*_{110}_*:V*_{411}_ *= 1*, the etchings of {110} and {411} simultaneously proceed. When {411} facet arrives point C, square mask starts to be undercut. The undercutting of strip goes on until {411} facet arrives point R. The overall etching depth along {100} is *H_j_*. *H_ct_* is the etching depth during the period when strip is undercut, *H_sq_* is the depth related to square mask, *H_d_* is the depth during the time from point C to R:

(7) $$H_{j}=H_{\mathrm{ct}}+H_{sq}-H_{d}=[0.5W_{t}\cos\alpha +L\sin\alpha +0.5W_{q}(\sin\alpha +\cos\alpha )-0.5W_{t}\sin\alpha ]\sin\beta \frac{V_{\left\{100\right\}}}{V_{\left\{411\right\}}}=0.13W_{t}+0.36L+0.46W_{q}$$

## 4. Results and Discussions

### 4.1. Fabrication Results

**Figure 4:**
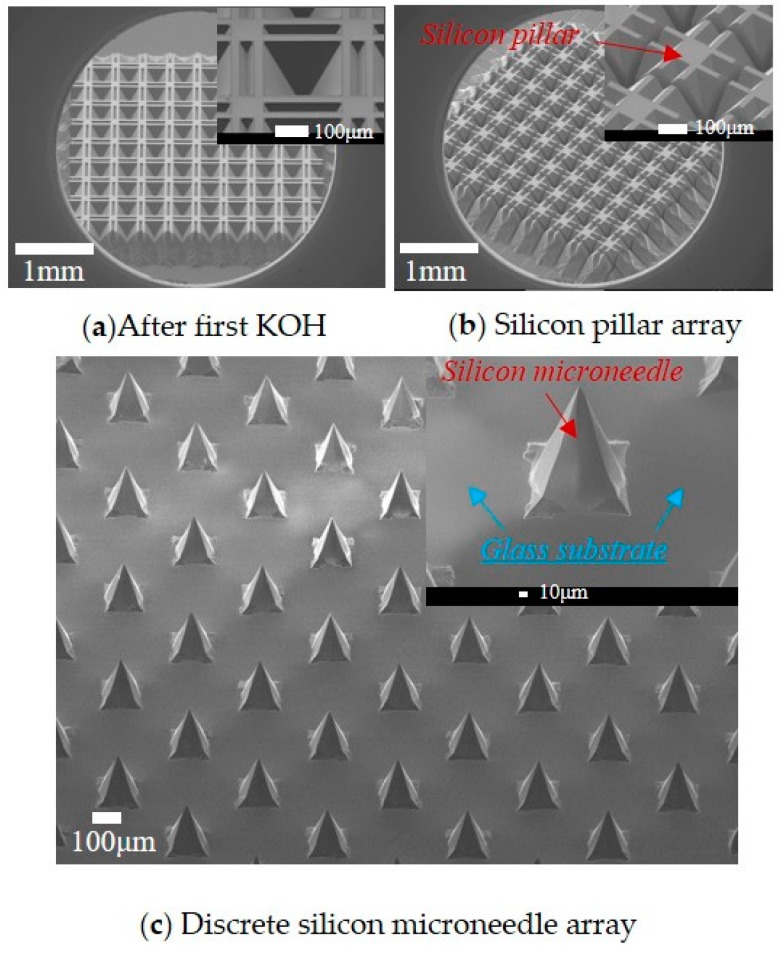
Fabrication process of microneedle array.

Three kinds of MNAs with different height are demonstrated, which are 190 μm, 270 μm, and 900 μm respectively. Here, MNA with 190 μm is taken as example to exhibit fabrication process. Figure 4 is obtained via scanning electron microscopy (SEM). Figure 4a demonstrates the silicon profile after 325 μm-thick silicon is firstly etched via KOH process. It could be noted that 75 μm-thick silicon is left in the margin region without Si_3_N_4_ mask. The inset shows the compensation strip is about 200 μm left, meaning that 900 μm-long strip is undercut. And {411} facet could be observed beneath the strip, as well as other step-shaped facets.

Then first KOH undercutting of the strip with width of 30 μm and length of 900 μm is considered. According to Equation (4), the corresponding etching depth *H_c_* = 333 μm, which is in accordance with practical value 325 μm. Figure 4b shows silicon pillar array with cross-like mask after dicing. The dimension of dicing groove is 40 μm-wide and 325 μm-deep, and the pitch is 340 μm. In order to form 9 × 9 array, 10 horizontal dicing and 10 vertical dicing are applied.

Figure 4c indicates discrete silicon MNA with height of 190 μm is fabricated after second KOH etching. Here, the pitch of microneedle is 340 um. The inset demonstrates that the microneedle is an octagonal pyramid comprising of eight inclining facet. As a matter of fact, similar MNA could be manufactured via once KOH undercutting of square mask. However, to manufacture 190 μm-height MNA, 500 μm-wide square mask is required, which means the pitch of microneedle would be more than 500 μm. Hence, it could be noted that KOH-dicing-KOH process is favorable to manufacture discrete silicon MNA with higher height-pitch ratio. According to Equation (6), when the dimension of cross-like mask is measured as: *W_q_* = 92 μm, *L* = 92 μm, *W_t_* = 22 μm, *H_j_* is figured out as 78 μm. In contrast, the etching depth in practice is 66 μm, which is smaller than theoretic value. The possible reasons may be the etching to SiO_2_ layer and inaccurate *V*_{411}_*:V*_{100}_. 

### 4.2. Facet of Octagonal Pyramid

**Figure 5:**
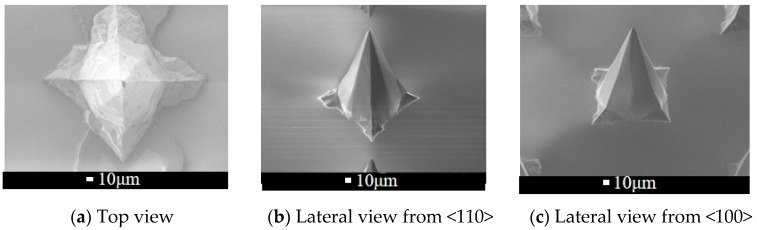
Top view and lateral view SEMs of microneedles.

As mentioned before, the etching could be divided into three parts: the upper inclining facet under mask is {411}, the middle one is mainly along {110} and lower one determines the final shape of microneedle, which is diverse in different condition. I. Silicon microneedles with {311} facets are fabricated under conditions of 80 °C and 40% KOH [19] as well as under conditions of 70 °C and 34% KOH [20]. Dizon proposed a fabrication method to form {411}-facet microneedles in condition of 83 °C and 33% KOH [21]. Wilke reported {321}-facet microneedles were realized under conditions of 79 °C and 29% KOH [22], which is very close to the conditions used in this paper. The angles measured from a top view (Figure 5a) and lateral view (Figure 5b,c) are compared with those of a theoretical {321}-facet pyramid, as shown in Table 2. Ultimately, the {321} facet is verified as the surface of octagonal pyramid microneedle.

### 4.3. MNAs with Diverse Height and Pitch

**Figure 6:**
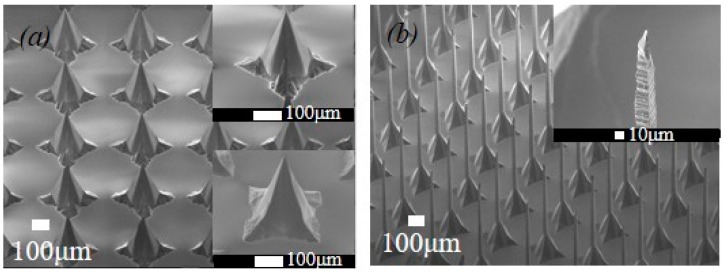
SEMs of Microneedle arrays with (**a**) 270 μm height and 420 μm pitch; (**b**) 900 μm height and 340 μm pitch.

Discrete MNAs with diverse height and pitch are also fabricated, as Figure 6 shows. MNAs with 270 μm-high and 420 μm-pitch are illustrated in Figure 6a, which accords with the aforementioned octagonal pyramid profile. After first KOH etching, *W_c_* = 40 μm, L = 880 μm, according to Equation (4), the corresponding etching depth *H_c_* = 329 μm, which is in accordance with the practical value of 320 μm. According to Equation (6), when the dimension of cross-like mask is measured as: *W_q_* = 143 μm, *L* = 106 μm, *W_t_* = 24 μm, *H_j_* is figured out as 107 μm. In contrast, the etching depth in practice is 96 μm, meaning that the estimation error is about 10%. The possible reasons may be the etching to SiO_2_ layer and inaccurate *V_{411}_:V_{100}_*, as mentioned before.

Figure 6b shows a MNA with 900 μm-high and 340 μm-pitch, which consists of slender tips, straight pillars and pyramid bases. As a matter of fact, the evolution of this microneedle could be observed in simulation, as Figure 3 showed. The upper inclining facets under mask constitute slender tips, the middle ones along {110} compose the straight pillars and the lower ones form the pyramidal base. It is obvious that this kind of high microneedle could not undergo the penetration force. Nevertheless, it could have potential in non-contact applications such as field emission.

### 4.4. Metallization on MNAs

**Figure 7:**
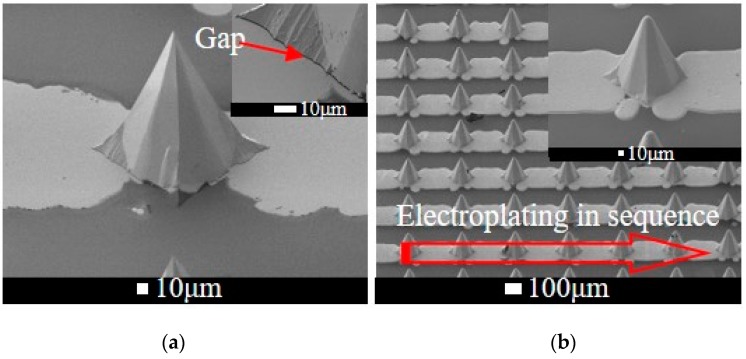
SEMs of microneedle arrays with (**a**) metal sputtering; (**b**) successive electroplating.

‘Metallization’ means to deposit and pattern a metal on the MNA, which makes it possible for electrical applications. It should be noted that there is a gap between discrete silicon microneedles and glass substrates, as in Figure 7. The gap in the range of several micrometers is inevitably caused due to KOH etching of the glass. However, the gap is fatal for the metallization because the thickness of routine metal sputtering is about several hundred nanometers and too small to cover the gap. Hence, a probable solution via successive electroplating is proposed. Specially, 150 nm Cr/Au is sputtered and patterned on the wafer. The metals on microneedle and glass are separated (Figure 7a).

Subsequently, electroplating starts from one side. The No.1 microneedle would be electroplated when the thickness of the metal line reaches up to the edge of microneedle and fills the gap. Then, the metal on microneedle is thickened, leading to connecting with adjacent metal line. Therefore, electroplating goes on in sequence and finally all the microneedles on this line are electroplated (Figure 7b). In fact, the metal is apt to be electroplated thicker on the edge. As a consequence, the gap could be quickly filled.

## 5. Conclusions

In this paper, an improved KOH-dicing-KOH process is proposed in order to fabricate discrete silicon MNAs with tunable height-pitch ratio. Compared with previously reported MNAs, their advantage lies in the discreteness and height-pitch ratio. Many efforts are made to demonstrate the formation of MNAs, including simulation, mathematical calculations and SEM experiments. In addition, the observed octagonal pyramids are confirmed to be composed of {321} facets. Further experiments indicate it has potential to produce diverse MNAs for versatile applications. The problem existing in metallization is solved via successive electroplating, which makes it feasible for microneedle electrode applications. In conclusion, the fabrication and analysis of these MNAs are demonstrated. Further research on the applications of these MNAs will be carried out.

## Figures and Tables

**Table 1:** Diverse microneedle arrays.

Source	Process	Material	Shape	Uniformity ^a^	Discreteness ^b^	Ratio of Height to Pitch
Kim [5]	Replication and curved deformation	Biodegradable polylactic acid (PLA)	Cone	fair	poor	350/950 = 0.37
Tu [6]	CO_2_ laser ablation and polymer molding	poly-lactic-co-glycolic acid (PLGA)	Cone	fair	poor	1179/500 = 2.36
Keum [7]	Polymer molding	Polycarprolactone (PCL)	Cone	fair	poor	700/900 = 0.78
Kusamori [8]	Micro-molding	Interferon α, polyvinyl alcohol (PVA)	Cone	fair	poor	800/900 = 0.89
Martin [9]	Low temperature vacuum deposition micromoulding	biodegradable sugar glass	Octagonal pyramid	good	poor	250/1000 = 0.25
Arai [10]	Replica molding	SU-8	candle-shaped	good	poor	1000/830 = 1.20
Ren [3]	Thermal drawing	PLGA	Hill-like	good	fair	500/1000 = 0.5
Stavrinidis [11]	Backside inadequate exposure	SU-8	Cone	poor	fair	500/650 = 0.77
Vinayakumar [12]	Electron discharge machining (EDM)	stainless steel	Hollow truncated Cone	fair	poor	300/500 = 0.6
Tezuka [13]	Electrochemical etching and insertion	Titanium	Cone	fair	good	800/2500 = 0.32
Yoon [15]	DRIE + HNA	Silicon	Cone	good	fair	380/340 = 1.12
Li [16]	DRIE + HNA	silicon	Cone	good	fair	150/200 = 0.75
Deng [2]	Dicing + HNA	silicon	rectangular pyramid	good	fair	200/90 = 2.22
Lin [17]	2 Stage etch	Silicon	self-stabilized diamond-shaped	good	fair	250/200 = 1.25
Wang [4]	Dry etch	Silicon	nipple-shaped	fair	good	80/450 = 0.18
O’Mahony [18]	One stage KOH	Silicon	Octagonal pyramid	good	good	300/1200 = 0.25
This work	KOH-dicing-KOH	Silicon	Octagonal pyramid	good	good	Bigger than 0.56, up to 2.65

^a^ The uniformity degree is judged from the reported figures, which is divided into three ranks. Higher rank means the profile and height of microneedles are more identical. ^b^ The discreteness degree is judged from the fabrication process. The more possible the microneedle and substrate are made of different material, the higher rank it would be.

**Table 2:** Angles from different view.

	Top View		Lateral View	
	Internal Angle 1 ^a^	Internal Angle 2 ^a^	γ_<110>_^b^	γ_<100>_^b^
Calculated {321}-facet pyramid	143.1°	126.9°	37.2°	36.7°
Measure from SEM	136.8° ± 5.4°	133.8° ± 3.8°	39.4°	37.8°

^a^ The internal angles could be measured directly from top view SEM. **^b^** The cone angles of pyramid observed from <110> and <100> are γ_<110>_ and γ_<100>_, respectively, which could be calculated from lateral view SEM. The angle between SEM probe and the wafer is *ψ* (here is 45°), and the measured cone angles in SEM is *θ*. Hence, γ_<110>_ and γ_<100>_ are determined by $\tan\frac{\gamma }{2}=\cos\psi \times \tan\frac{\theta }{2}$.
